# 1-(5,7-Dihy­droxy-2,2-dimethylchroman-6-yl)ethanone

**DOI:** 10.1107/S1600536811047982

**Published:** 2011-11-23

**Authors:** Matthew P. Akerman, Zimbili Mkhize, Fanie R. van Heerden

**Affiliations:** a School of Chemistry, University of KwaZulu-Natal, Private Bag X01, Scottsville, 3209, Pietermaritzburg, South Africa

## Abstract

In the title mol­ecule, C_13_H_16_O_4_, the pyran ring is in a half-chair conformation. There is an intra­molecular hydrogen bond involving the ketone O atom and an H atom of a phenol group which forms an *S*(6) ring. The ketone O atom is also involved in an inter­molecular hydrogen bond with a different phenolic H atom of a symmetry-related mol­ecule, forming *C*(6) chains along the *c*-axis direction.

## Related literature

For applications of the title compound, see: Kraus *et al.* (2011 ▶); Basabe *et al.* (2010 ▶). For hydrogen-bond motifs, see: Bernstein *et al.* (1995 ▶). For a related structure, see: Chakkaravarthi *et al.* (2007 ▶).
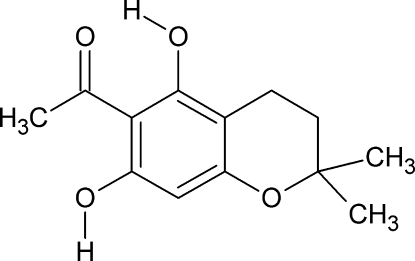


## Experimental

### 

#### Crystal data

C_13_H_16_O_4_
*M* *_r_* = 236.26
Tetragonal, 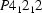

*a* = 10.5677 (2) Å
*c* = 21.4244 (5) Å
*V* = 2392.6 (1) Å^3^
*Z* = 8
Mo *K*α radiation
μ = 0.10 mm^−1^
*T* = 298 K
0.6 × 0.4 × 0.4 mm

#### Data collection

Oxford Diffraction Xcalibur 2 CCD diffractometer
Absorption correction: multi-scan (*SORTAV*; Blessing, 1995 ▶) *T*_min_ = 0.955, *T*_max_ = 0.962
26011 measured reflections
2366 independent reflections
2046 reflections with *I* > 2σ(*I*)
*R*_int_ = 0.047

#### Refinement

*R*[*F* ^2^ > 2σ(*F* ^2^)] = 0.032
*wR*(*F* ^2^) = 0.091
*S* = 1.08
2366 reflections
166 parameters
H atoms treated by a mixture of independent and constrained refinement
Δρ_max_ = 0.12 e Å^−3^
Δρ_min_ = −0.11 e Å^−3^
Absolute structure: Flack (1983 ▶), 931 Friedel pairs
Flack parameter: 0.7 (11)

### 

Data collection: *CrysAlis CCD* (Oxford Diffraction, 2008 ▶); cell refinement: *CrysAlis CCD*; data reduction: *CrysAlis RED* (Oxford Diffraction, 2008 ▶); program(s) used to solve structure: *SHELXS97* (Sheldrick, 2008 ▶); program(s) used to refine structure: *SHELXL97* (Sheldrick, 2008 ▶); molecular graphics: *WinGX* (Farrugia, 1999 ▶); software used to prepare material for publication: *publCIF* (Westrip, 2010 ▶).

## Supplementary Material

Crystal structure: contains datablock(s) I. DOI: 10.1107/S1600536811047982/lh5372sup1.cif

Structure factors: contains datablock(s) I. DOI: 10.1107/S1600536811047982/lh5372Isup2.hkl

Supplementary material file. DOI: 10.1107/S1600536811047982/lh5372Isup3.mol

Supplementary material file. DOI: 10.1107/S1600536811047982/lh5372Isup4.cml

Additional supplementary materials:  crystallographic information; 3D view; checkCIF report

## Figures and Tables

**Table 1:** Hydrogen-bond geometry (Å, °)

*D*—H⋯*A*	*D*—H	H⋯*A*	*D*⋯*A*	*D*—H⋯*A*
O2—H102⋯O4^i^	0.97 (2)	1.77 (2)	2.737 (2)	179 (1)
O3—H103⋯O4	0.86 (2)	1.71 (2)	2.501 (2)	151 (2)

Symmetry code: (i) 
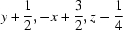
.

**Fig. 1.**
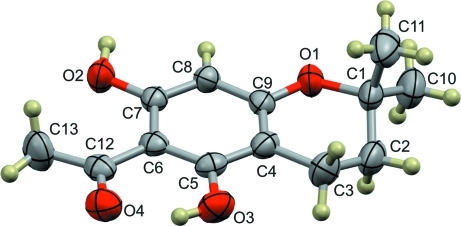
The molecular structure of the title compound with 50% probability ellipsoids. Hydrogen atoms have been rendered as spheres of arbitrary radius.

**Fig. 2.**
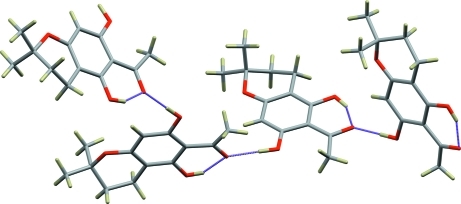
Part of a hydrogen bonded (dashed lines) chain along [001].

## supplementary crystallographic information

### Comment

The title compound was synthesized as an intermediate in the preparation of flavonoids or other phenolic derivatives and an intermediate for an anti-HIV chromanone (Kraus *et al.*, 2011). It has also been obtained as a side product in the preparation of prenylated flavonoids with antitumour activity (Basabe *et al.*, 2010). The molecular structure of the title compound is shown in Fig. 1. The pyran ring is in a half-chair conformation. There are two types of hydrogen bonds, one intramolecular and one intermolecular. The intramolecular O3—H103···O4 hydrogen bond forms an S(6) ring motif (Bernstein *et al.*, 1995). This hydrogen bond motif is common to molecules which contain derivatized (2-hydroxyphenyl)ethanone structures (Chakkaravarthi *et al.*, 2007). In addition to the intramolecular hydrogen bonding, there is an intermolecular hydrogen bond between the phenolic group and the ketone O atom of an adjacent molecule. This O2—H102···O4^i^ (see Table 1 for symmetry code) hydrogen bond links the molecules to form infinite one-dimensional C(6) chains parallel to the *c* axis (base vector [0 0 1]). The same ketone oxygen atom therefore accepts two hydrogen bonds, one intermolecular and one intramolecular. The hydrogen bond lengths and bond angles are summarized in Table 1. Fig.2 depicts both the intermolecular and intramolecular hydrogen bonds. The length of intermolecular hydrogen bond is 0.303 Å shorter than the sum of the van der Waals radii. Although the length of hydrogen bonds does not necessarily correlate linearly with bond strength, due to packing constraints in the lattice, it is probable that this very short bond is moderate to strong. This is especially likely considering that the bond angle very closely approaches ideality.

### Experimental

To a solution of 6-hydroxy-2,4-dimethoxymethyloxy-3-prenylacetophenone (80 mg, 0.25 mmol) in methanol (20 ml) was added 1.0 *M* HCl (6 ml). The reaction mixture was refluxed for 1 h before cooling. The solvent was evaporated and the residue purified by column chromatography using hexane:ethyl acetate: 2:1 to afford 1-(5,7-dihydroxy-2,2-dimethylchroman-6-yl)ethanone as yellow crystals (10 mg, 17%): mp 501–502 K;

### Refinement

The positions of all hydrogen atoms bonded to C atoms were calculated using the standard riding model of *SHELXL97* (Sheldrick, 2008) with C—H(aromatic) and C—H (methylene) distances of 0.93 Å and *U*_iso_ = 1.2 *U*_eq_, and *C*—*H*(methyl) distances of 0.96 Å and *U*_iso_ = 1.5*U*_eq_. The phenolic hydrogen atoms were located in the difference Fourier map and allowed to refine isotropically.

### Crystal data

**Table:**

C_13_H_16_O_4_	*D*_x_ = 1.312 Mg m^−^^3^
*M**_r_* = 236.26	Mo *K*α radiation, λ = 0.71073 Å
Tetragonal, *P*4_1_2_1_2	Cell parameters from 2366 reflections
Hall symbol: P 4abw 2nw	θ = 2.9–26.0°
*a* = 10.5677 (2) Å	µ = 0.10 mm^−^^1^
*c* = 21.4244 (5) Å	*T* = 298 K
*V* = 2392.6 (1) Å^3^	Needle, colourless
*Z* = 8	0.6 × 0.4 × 0.4 mm
*F*(000) = 1008	

### Data collection

**Table:**

Oxford Diffraction Xcalibur 2 CCD diffractometer	2366 independent reflections
Radiation source: fine-focus sealed tube	2046 reflections with *I* > 2σ(*I*)
graphite	*R*_int_ = 0.047
Detector resolution: 8.4190 pixels mm^-1^	θ_max_ = 26.0°, θ_min_ = 2.9°
ω scans at fixed θ angles	*h* = −13→13
Absorption correction: multi-scan (*SORTAV*; Blessing, 1995)	*k* = −13→13
*T*_min_ = 0.955, *T*_max_ = 0.962	*l* = −26→26
26011 measured reflections	

### Refinement

**Table:**

Refinement on *F*^2^	Hydrogen site location: inferred from neighbouring sites
Least-squares matrix: full	H atoms treated by a mixture of independent and constrained refinement
*R*[*F*^2^ > 2σ(*F*^2^)] = 0.032	*w* = 1/[σ^2^(*F*_o_^2^) + (0.0593*P*)^2^ + 0.0612*P*] where *P* = (*F*_o_^2^ + 2*F*_c_^2^)/3
*wR*(*F*^2^) = 0.091	(Δ/σ)_max_ = 0.001
*S* = 1.08	Δρ_max_ = 0.12 e Å^−^^3^
2366 reflections	Δρ_min_ = −0.11 e Å^−^^3^
166 parameters	Extinction correction: *SHELXL*, Fc^*^=kFc[1+0.001xFc^2^λ^3^/sin(2θ)]^-1/4^
0 restraints	Extinction coefficient: 0.0094 (18)
Primary atom site location: structure-invariant direct methods	Absolute structure: Flack (1983), 931 Friedel pairs
Secondary atom site location: difference Fourier map	Flack parameter: 0.7 (11)

### Special details

**Table:**

Experimental. ^1^H NMR (400 MHz, CD_3_OD) 1.31 (2*x* 3H, *s*, C(CH_3_)_2_), 1.78 (2*H*, *t, J* = 6.7 Hz, CH_2_), 2.55 (2*H*, *t, J* = 6.7 Hz, CH_2_), 2.62 (3*H*, *s*, COCH_3_), 5.77 (1*H*, *s*, ArH); ^13^C NMR 15.6 (C(CH_3_)_2_), 25.6 (2 × CH_2_), 31.3 (C(CH_3_)_2_), 31.8 (COCH_3_), 75.3 (C(CH_3_)_2_, 94.5 (C-5), 99.9 (C-1), 104.2 (C-3), 160.0, 160.9, 163.3 (C-2,4,6), 203.4 (COCH_3_); ESITOFMS, *m/z* 259.0945 [*M*+Na]^+^ (calc. for C_13_H_16_NaO_4_ 259.0946); IR (KBr) υ 2961 2918 2872 1654 1611 1433 1159 cm^-1^.
Geometry. All s.u.'s (except the s.u. in the dihedral angle between two l.s. planes) are estimated using the full covariance matrix. The cell s.u.'s are taken into account individually in the estimation of s.u.'s in distances, angles and torsion angles; correlations between s.u.'s in cell parameters are only used when they are defined by crystal symmetry. An approximate (isotropic) treatment of cell s.u.'s is used for estimating s.u.'s involving l.s. planes.
Refinement. Refinement of *F*^2^ against ALL reflections. The weighted *R*-factor *wR* and goodness of fit *S* are based on *F*^2^, conventional *R*-factors *R* are based on *F*, with *F* set to zero for negative *F*^2^. The threshold expression of *F*^2^ > 2σ(*F*^2^) is used only for calculating *R*-factors(gt) *etc*. and is not relevant to the choice of reflections for refinement. *R*-factors based on *F*^2^ are statistically about twice as large as those based on *F*, and *R*- factors based on ALL data will be even larger.

### Fractional atomic coordinates and isotropic or equivalent isotropic displacement parameters (Å^2^)

**Table:**

	*x*	*y*	*z*	*U*_iso_*/*U*_eq_	
H102	1.1657 (18)	0.6538 (18)	0.1786 (9)	0.064 (5)*	
H103	0.794 (2)	0.877 (2)	0.3250 (10)	0.075 (7)*	
O1	0.94129 (10)	0.98280 (11)	0.09038 (5)	0.0540 (3)	
O2	1.11831 (12)	0.66838 (11)	0.21655 (5)	0.0563 (3)	
C9	0.93675 (14)	0.92341 (14)	0.14598 (6)	0.0410 (3)	
O3	0.77624 (12)	0.92148 (12)	0.29235 (6)	0.0619 (3)	
C7	1.03346 (13)	0.76254 (13)	0.20884 (7)	0.0390 (3)	
O4	0.87361 (12)	0.75511 (12)	0.36008 (5)	0.0611 (4)	
C8	1.02635 (13)	0.82754 (14)	0.15389 (7)	0.0403 (3)	
H8	1.0815	0.8079	0.1215	0.048*	
C4	0.85141 (14)	0.95417 (14)	0.19248 (7)	0.0452 (3)	
C6	0.95010 (13)	0.79073 (13)	0.25928 (6)	0.0397 (3)	
C12	0.95429 (15)	0.72843 (14)	0.31906 (7)	0.0468 (4)	
C5	0.86024 (13)	0.88832 (13)	0.24823 (7)	0.0427 (3)	
C1	0.83994 (17)	1.07167 (15)	0.07346 (8)	0.0553 (4)	
C2	0.79788 (18)	1.14369 (17)	0.12997 (8)	0.0657 (5)	
H2A	0.7282	1.1988	0.1186	0.079*	
H2B	0.8670	1.1966	0.1444	0.079*	
C3	0.75608 (18)	1.05776 (18)	0.18283 (9)	0.0670 (5)	
H3A	0.7474	1.1067	0.2209	0.080*	
H3B	0.6743	1.0211	0.1730	0.080*	
C13	1.0503 (2)	0.6319 (2)	0.33569 (9)	0.0717 (6)	
H13A	1.0384	0.6062	0.3783	0.108*	
H13B	1.1334	0.6671	0.3308	0.108*	
H13C	1.0412	0.5598	0.3088	0.108*	
C10	0.9031 (2)	1.15856 (19)	0.02626 (9)	0.0774 (6)	
H10A	0.9746	1.1991	0.0453	0.116*	
H10B	0.8437	1.2216	0.0127	0.116*	
H10C	0.9307	1.1098	−0.0090	0.116*	
C11	0.7364 (2)	0.9942 (2)	0.04359 (11)	0.0801 (6)	
H11A	0.7687	0.9537	0.0068	0.120*	
H11B	0.6672	1.0485	0.0325	0.120*	
H11C	0.7076	0.9311	0.0725	0.120*	

### Atomic displacement parameters (Å^2^)

**Table:**

	*U*^11^	*U*^22^	*U*^33^	*U*^12^	*U*^13^	*U*^23^
O1	0.0605 (7)	0.0550 (7)	0.0466 (6)	0.0166 (5)	−0.0064 (5)	0.0060 (5)
O2	0.0642 (7)	0.0578 (7)	0.0469 (6)	0.0220 (6)	0.0037 (5)	0.0064 (5)
C9	0.0420 (8)	0.0391 (8)	0.0418 (7)	0.0004 (6)	−0.0102 (6)	−0.0035 (6)
O3	0.0626 (7)	0.0610 (8)	0.0621 (8)	0.0078 (6)	0.0188 (7)	−0.0060 (6)
C7	0.0381 (7)	0.0363 (7)	0.0427 (7)	0.0005 (6)	−0.0055 (6)	−0.0035 (6)
O4	0.0725 (8)	0.0614 (7)	0.0495 (6)	−0.0088 (6)	0.0142 (6)	0.0021 (5)
C8	0.0384 (7)	0.0433 (8)	0.0393 (7)	0.0037 (6)	−0.0016 (5)	−0.0030 (6)
C4	0.0408 (8)	0.0400 (8)	0.0548 (8)	0.0043 (6)	−0.0045 (7)	−0.0072 (6)
C6	0.0425 (8)	0.0359 (7)	0.0406 (7)	−0.0070 (6)	−0.0024 (6)	−0.0052 (6)
C12	0.0533 (9)	0.0437 (8)	0.0433 (8)	−0.0129 (7)	−0.0015 (7)	−0.0023 (7)
C5	0.0388 (8)	0.0403 (7)	0.0492 (8)	−0.0038 (6)	0.0028 (6)	−0.0100 (6)
C1	0.0624 (10)	0.0412 (8)	0.0622 (10)	0.0110 (7)	−0.0207 (8)	0.0042 (7)
C2	0.0660 (11)	0.0489 (9)	0.0822 (12)	0.0191 (9)	−0.0169 (10)	−0.0018 (9)
C3	0.0609 (11)	0.0643 (11)	0.0758 (12)	0.0237 (9)	−0.0025 (9)	−0.0044 (9)
C13	0.0795 (13)	0.0825 (14)	0.0532 (10)	0.0105 (10)	0.0037 (9)	0.0208 (9)
C10	0.0952 (15)	0.0560 (11)	0.0811 (13)	0.0109 (11)	−0.0145 (11)	0.0178 (10)
C11	0.0806 (14)	0.0621 (11)	0.0975 (15)	0.0070 (11)	−0.0408 (12)	−0.0017 (11)

### Geometric parameters (Å, °)

**Table:**

O1—C9	1.3471 (18)	C1—C2	1.497 (2)
O1—C1	1.4699 (18)	C1—C11	1.509 (3)
O2—C7	1.3496 (17)	C1—C10	1.520 (3)
O2—H102	0.97 (2)	C2—C3	1.517 (3)
C9—C4	1.383 (2)	C2—H2A	0.9700
C9—C8	1.397 (2)	C2—H2B	0.9700
O3—C5	1.3432 (18)	C3—H3A	0.9700
O3—H103	0.86 (2)	C3—H3B	0.9700
C7—C8	1.365 (2)	C13—H13A	0.9600
C7—C6	1.426 (2)	C13—H13B	0.9600
O4—C12	1.2566 (19)	C13—H13C	0.9600
C8—H8	0.9300	C10—H10A	0.9600
C4—C5	1.386 (2)	C10—H10B	0.9600
C4—C3	1.502 (2)	C10—H10C	0.9600
C6—C5	1.422 (2)	C11—H11A	0.9600
C6—C12	1.441 (2)	C11—H11B	0.9600
C12—C13	1.482 (2)	C11—H11C	0.9600
			
C9—O1—C1	119.33 (12)	C1—C2—C3	112.68 (15)
C7—O2—H102	111.0 (11)	C1—C2—H2A	109.1
O1—C9—C4	123.42 (14)	C3—C2—H2A	109.1
O1—C9—C8	114.90 (12)	C1—C2—H2B	109.1
C4—C9—C8	121.68 (13)	C3—C2—H2B	109.1
C5—O3—H103	106.8 (14)	H2A—C2—H2B	107.8
O2—C7—C8	120.88 (13)	C4—C3—C2	110.10 (15)
O2—C7—C6	118.15 (13)	C4—C3—H3A	109.6
C8—C7—C6	120.97 (13)	C2—C3—H3A	109.6
C7—C8—C9	120.46 (13)	C4—C3—H3B	109.6
C7—C8—H8	119.8	C2—C3—H3B	109.6
C9—C8—H8	119.8	H3A—C3—H3B	108.2
C9—C4—C5	117.34 (13)	C12—C13—H13A	109.5
C9—C4—C3	120.64 (15)	C12—C13—H13B	109.5
C5—C4—C3	121.99 (14)	H13A—C13—H13B	109.5
C5—C6—C7	115.98 (13)	C12—C13—H13C	109.5
C5—C6—C12	120.00 (13)	H13A—C13—H13C	109.5
C7—C6—C12	124.01 (13)	H13B—C13—H13C	109.5
O4—C12—C6	119.89 (15)	C1—C10—H10A	109.5
O4—C12—C13	116.79 (14)	C1—C10—H10B	109.5
C6—C12—C13	123.32 (14)	H10A—C10—H10B	109.5
O3—C5—C4	115.54 (14)	C1—C10—H10C	109.5
O3—C5—C6	120.90 (14)	H10A—C10—H10C	109.5
C4—C5—C6	123.56 (13)	H10B—C10—H10C	109.5
O1—C1—C2	109.98 (12)	C1—C11—H11A	109.5
O1—C1—C11	106.62 (13)	C1—C11—H11B	109.5
C2—C1—C11	113.80 (18)	H11A—C11—H11B	109.5
O1—C1—C10	103.32 (15)	C1—C11—H11C	109.5
C2—C1—C10	111.17 (15)	H11A—C11—H11C	109.5
C11—C1—C10	111.33 (16)	H11B—C11—H11C	109.5
			
C1—O1—C9—C4	−9.6 (2)	C9—C4—C5—O3	179.59 (12)
C1—O1—C9—C8	170.94 (13)	C3—C4—C5—O3	1.4 (2)
O2—C7—C8—C9	178.90 (13)	C9—C4—C5—C6	−1.1 (2)
C6—C7—C8—C9	0.0 (2)	C3—C4—C5—C6	−179.29 (14)
O1—C9—C8—C7	178.31 (12)	C7—C6—C5—O3	179.24 (13)
C4—C9—C8—C7	−1.2 (2)	C12—C6—C5—O3	−1.8 (2)
O1—C9—C4—C5	−177.76 (13)	C7—C6—C5—C4	0.0 (2)
C8—C9—C4—C5	1.7 (2)	C12—C6—C5—C4	178.93 (13)
O1—C9—C4—C3	0.5 (2)	C9—O1—C1—C2	36.82 (19)
C8—C9—C4—C3	179.93 (15)	C9—O1—C1—C11	−87.00 (18)
O2—C7—C6—C5	−178.34 (12)	C9—O1—C1—C10	155.56 (14)
C8—C7—C6—C5	0.6 (2)	O1—C1—C2—C3	−55.9 (2)
O2—C7—C6—C12	2.7 (2)	C11—C1—C2—C3	63.6 (2)
C8—C7—C6—C12	−178.32 (13)	C10—C1—C2—C3	−169.70 (14)
C5—C6—C12—O4	4.3 (2)	C9—C4—C3—C2	−19.4 (2)
C7—C6—C12—O4	−176.84 (14)	C5—C4—C3—C2	158.76 (15)
C5—C6—C12—C13	−175.88 (16)	C1—C2—C3—C4	47.0 (2)
C7—C6—C12—C13	3.0 (2)		

### Hydrogen-bond geometry (Å, °)

**Table:**

*D*—H···*A*	*D*—H	H···*A*	*D*···*A*	*D*—H···*A*
O2—H102···O4^i^	0.97 (2)	1.77 (2)	2.737 (2)	179 (1)
O3—H103···O4	0.86 (2)	1.71 (2)	2.501 (2)	151 (2)

Symmetry codes: (i) *y*+1/2, −*x*+3/2, *z*−1/4.
